# Inequitable access to an outpatient parenteral antimicrobial therapy service: linked cross-sectional study

**DOI:** 10.1186/s12939-020-01261-w

**Published:** 2020-09-01

**Authors:** Colin Sumpter, Clark D. Russell, Claire Mackintosh

**Affiliations:** 1 Department of Public Health and Policy, NHS Lothian, Waverley Gate, Edinburgh, UK; 2 The University of Edinburgh Centre for Inflammation Research, Edinburgh, Edinburgh, UK; NHS Lothian, Regional Infectious Diseases Unit, Edinburgh, UK; 3 NHS Lothian, Regional Infectious Diseases Unit, Edinburgh, UK

**Keywords:** Socioeconomic factors, Health Equity, Outpatients, Anti-Bacterial Agents

## Abstract

**Study aim:**

To assess whether Outpatient Parenteral Antimicrobial Therapy (OPAT) is provided equitably across gender and social groups in a tertiary care setting.

**Background:**

OPAT is a widely used and growing approach in high income countries to early discharge or admission avoidance for patients requiring intravenous antimicrobials. There is however a risk that equitable access to healthcare could be eroded unintentionally by expansion of outpatient or ambulatory approaches such as this. Anecdotal evidence in our service, and from published studies, have identified a gender and social group equity gap in outpatient services.

**Methods:**

Service data on inpatient cellulitis episodes over a seven-year period were matched to OPAT referral data to create a retrospective cross-sectional linked dataset. All individuals admitted from 2012 to 2017 inclusive for a primary diagnosis of cellulitis were included: 6295 admissions of 4944 individuals. Demographics, number of co-morbidities, length of hospital stay, number of admissions, distance from OPAT unit and Scottish Index of Multiple Deprivation (SIMD; as a metric of deprivation) were recorded. Adjusted odds of a referral to OPAT across SIMD quintiles and for females compared to males were calculated using multiple logistic regression.

**Results:**

Inequitable access to OPAT was identified. Deprivation was negatively associated with likelihood of OPAT referral. Inpatients from the most affluent SIMD quintile were more than twice as likely to have received an OPAT referral compared to those resident in the most deprived quintile (adjusted OR 2.08, 95% CI: 1.60–2.71, *p* <  0.0001). Women were almost a third less likely to receive an OPAT referral than men (adjusted OR 0.69, 95% CI: 0.58 to 0.82, *p* <  0.001). Results were adjusted for age, number of co-morbidities, admissions, length of stay, distance from nearest OPAT unit, time since first admission, deprivation and gender.

**Conclusions:**

OPAT services and other ambulatory care programmes should routinely evaluate the equity of their service provision and consider how they can reduce any identified imbalance. It is a critical responsibility of service planning to ensure an inequitable system does not develop, with those least able to access ambulatory care dispossessed of the associated benefits.

## Background

Outpatient Parenteral Antimicrobial Therapy (OPAT) allows clinically stable patients to be discharged from hospital and attend a dedicated outpatient unit to receive intravenous antimicrobials and ongoing clinical assessment [1]. OPAT is a safe and efficacious modality for managing infection in an outpatient or home setting [2]. OPAT is considered to be significantly cost saving to overall health budgets through facilitating early discharge and avoidance of admission to inpatient settings [3].

The most commonly treated infections are skin and soft tissue infections such as cellulitis but a wide range of other infections can be effectively treated in OPAT [1, 3, 4]. The OPAT approach is growing in the UK [1] and globally [2]. In the UK this has been driven in part by the potential for cost saving and also as part of the drive to provide health services closer to patient homes [5]. Nationally the good practice recommendations in the UK suggest that OPAT should always be offered as an alternative to inpatient care and that patients should be able to choose between these options [1]..

There is a risk that equitable access to healthcare could be eroded by expansion of outpatient or ambulatory approaches. Health inequity is defined as differences in healthcare that are avoidable, unnecessary and unjust [6]. They can arise when patient access is provided not due to the ability to benefit from a service, but from the ability to obtain it. The equitable delivery of health services is a founding principle and remains a cornerstone of the National Health Service (NHS) [7] and is a stated aim of many healthcare systems across the world [8].

High quality international review level evidence has shown that socioeconomic position is a clear determinant of health care use across a wide range of specialities and interventions with the wealthy receiving more care than the poor, inverse to their need [9, 10]. Due to the free provision of healthcare at the point of need in the UK the context is different, but there is still evidence that when need and uptake are compared the NHS often favours those from wealthier areas compared to those from deprived areas [11–13]. In addition the poor suffer disproportionately from ambulatory care sensitive emergency admissions, such as those treated by OPAT [14].

The relationship between gender and outpatient services in high income countries is more complex. A recent systematic review found patient gender to be amongst the main barriers to uptake [15]. But the direction of this effect is not always clear with some studies finding men less likely to attend [16], and others women [17]. Studying the specific challenges and context of each outpatient service is vital to understanding these inequities.

Anecdotal observations during clinical practice in OPAT led to our interest in studying our own service along these lines. Our motivation to complete this study was both values based and financial. Understanding how social and gender determinants of health not only influence health outcomes but are themselves perpetuated by the organisation of resources is of significant importance for reasons of both social justice and cost-effectiveness of services. As ambulatory care continues to increase it will be an important challenge for healthcare providers to ensure they do not further exacerbate existing inequities.

To better understand this, we set out to assess the equity of access to the NHS Lothian OPAT service by comparing those who had ever been referred to the service, to those who had not, in a large eligible inpatient population: those admitted due to cellulitis. Equity of referral on the basis of gender and socio-economic deprivation were the focus of this investigation.

NHS Lothian is a health board in Scotland serving a population of around 900,000 people. NHS Lothian’s OPAT service has been in operation since 2011, initially in a central Edinburgh hospital with the addition in 2013 of a satellite peripheral centre to the west of the city centre. The service primarily operates a hospital-based service with around one third of patients trained to self-administer treatment at home. It is staffed by infectious disease doctors and nurses and offers a seven-day service.

## Methods

Admissions data was extracted for all inpatient admissions of NHS Lothian residents for cellulitis between 2012 and 2017 inclusive. Cellulitis was defined as a primary reason for admission ICD10 code commencing L03 (Cellulitis and acute lymphangitis) [18]. The OPAT service accepts referrals for patients aged 13 and above so this age cut-off was used.

Analysis was undertaken on the basis of individuals rather than admissions. The total number of admissions for cellulitis during the study period, the total number of co-morbidities during those specific admissions and the total length of stay arising from those admissions were calculated for each individual. Age at admission was derived from date of birth. Distance from OPAT unit was calculated by Euclidean distance (‘as the crow flies’) using postcode co-ordinates from Code Point Open. Age was divided into three equal groups (tertiles) with natural breakpoints to create a categorical variable.

Gender and Scottish Index of Multiple Deprivation (SIMD) quintile of residence were included as measures of equity. SIMD is an area based measure of relative deprivation that ranks small areas (called data zones) of Scotland from most deprived (ranked 1) to least deprived (ranked 6976) using a composite deprivation score that takes into account income, employment, education, health, access to services, crime and housing [19]. SIMD was used as the only available measure of deprivation of this historic cohort, SIMD is widely used as a measure of deprivation in equity research in Scotland. In this study national quintiles were used in the analysis.

Population data for denominators of rates were calculated using mid-2017 Small Area Population Estimates for 2011 data zones produced by National Records of Scotland [20].

The primary outcome was to have had any contact or referral with the NHS Lothian OPAT service over the period 2012 to 2017 inclusive (referred to in this article as being ‘known to OPAT’). Matching was done on the basis of the Case Reference Number (CRN) of the admissions data to the CRN of the OPAT referrals data stored on the electronic patient record (EPR; ‘TRAK care’). The CRN is a unique health record identifier used in secondary care in Scotland. The match was quality assured by independently matching the admissions data with a service-maintained database which is distinct from the EPR.

Descriptive statistics were calculated for cellulitis admissions including rates of admission per 10,000 population across demographic groups. A univariate comparison of individuals who had been referred to OPAT to those who had not been referred was carried out. The adjusted odds of a referral to OPAT across the SIMD quintiles, and the odds for females compared to males, were calculated. These multivariate odds ratios were computed using logistic regression. Odds ratios were adjusted for age, gender, SIMD, distance from nearest OPAT unit, time since first admission, total admissions, total co-morbidities and total length of stay over the period. Analysis was undertaken using R [21].

The datasets generated and/or analysed during the current study are not publicly available as they comprise confidential identifiable patient level data. The data may be available from the corresponding author on reasonable request to persons suitably authorised to view the data.

Data governance was practiced in accordance with the Caldicott principles. Ethical approval was assessed through the NHS Health Research Authority Decision Tool [22]. As this was a retrospective analysis of routine service data, no formal ethical approval was required.

## Results

Six thousand two hundred ninety-five inpatient admissions of 4944 individuals (aged ≥13 years) with a primary diagnosis of cellulitis were identified in NHS Lothian between 2012 and 2017 inclusive. Gender and age data were complete but individuals with no valid postcode were excluded from further analysis (*n* = 104). Distance from nearest OPAT unit was unavailable for a small number of individuals and these were excluded from the multivariate analysis (*n* = 42). Where individuals were identified as having different postcodes for subsequent admissions during the period, the postcode given at first admission was used (*n* = 59). A small number of individuals (*n* = 29) were present in the OPAT database and not the EPR, and a small number of EPR referrals were not recorded on the OPAT database (*n* = 69). It was decided that presence in either database merited inclusion in the analysis. Ultimately 4944 individuals were included in the univariate analysis and 4902 in the multivariate analysis.

This patient group required a total of 54,150 bed days over the period, an average of 9025 per year. There was a trend for increased admissions due to cellulitis with a relative increase of 16% between 2012 and 2017.

Across the whole cohort admitted around there were clear associations between cellulitis admission and age, gender with rates of admission significantly higher in men and older age groups. Details of the patient group are shown in Table 1. Where an individual had multiple admissions, data from their first admission was used.

**Table 1:** Individuals admitted for cellulitis, NHS Lothian, 2012 to 2017

	Individuals^a^ (*n* = 4944) n (%)	Rate of admission per 10,000 ^b^ (95% CI)
Gender		
*Male*	2642 (53%)	11.9 (10.9 to 13.1)
*Female*	2302 (47%)	9.7 (8.7 to 10.7)
Age Group		
*50 and under*	1607 (33%)	5.7 (5.1 to 6.4)
*51 to 70*	1648 (33%)	13.4 (11.8 to 15)
*71 and over*	1689 (34%)	31.0 (27.5 to 34.9)
SIMD Quintiles		
*(Most Deprived) 1*	845 (17%)	15.6 (13.1 to 18.4)
*2*	1354 (27%)	14.3 (12.5 to 16.3)
*3*	886 (18%)	11.0 (9.3 to 12.9)
*4*	749 (15%)	9.1 (7.6 to 10.9)
*(Least deprived) 5*	1110 (22%)	7.6 (6.5 to 8.7)

^a^ Individuals admitted at least once for a primary reason of cellulitis

^b^ Annual average number of individuals admitted at least once between 2012 and 2017 per 10,000 NHS Lothian residents aged 13+. 95% confidence interval obtained with Byar’s method

**Fig. 1:**
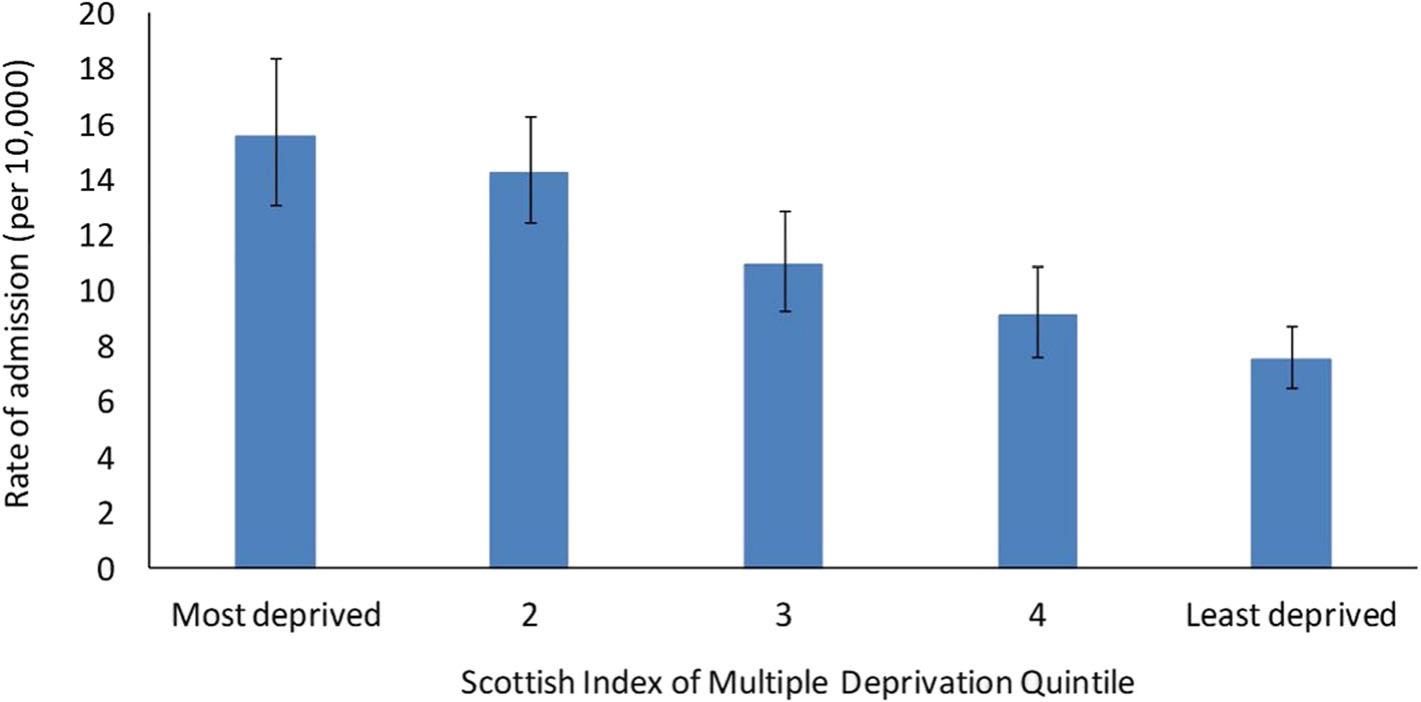
Rate of admission^1^ for cellulitis stratified by SIMD quintile per 10,000 population, NHS Lothian, 2012 to 2017. ^1^ Annual average number of individuals admitted at least once for a primary reason of cellulitis between 2012 and 2017 per 10,000 NHS Lothian residents aged 13+. Error bars show 95% confidence interval obtained with Byar’s method

Relative to Lothian’s resident population, a clear social gradient in admission for cellulitis was identified with individuals living in the most deprived quintile having a rate of admission twice that of those in the least deprived quintile (Fig. 1). Correlation between deprivation and cellulitis admission was significant (*p* <  0.0001, chi-squared test of linear trend). The median distance from home to nearest OPAT centre was 6.8 km (IQR: 3.8 km to 13.0 km). The median number of co-morbidities recorded was 3 (IQR: 1 to 7). 82% of individuals had only one admission over the period, 13% had 2 and the remaining 5% had more than 2, with a maximum of 12 admissions. The median length of stay for each individual was 4 days (IQR: 2 to 10 days).

Fifteen percent of individuals admitted due to cellulitis were identified as having been referred to or having accessed OPAT during the same period (729 individuals, 1140 admissions). Univariate analysis identified that variables likely to be associated with the severity of cellulitis (age, number of co-morbidities and length of stay) were all negatively associated with OPAT referral, as expected (Table 2). Importantly, equity of access variables were also associated with OPAT referral. Patients who were more deprived or female were less likely to be referred. The full univariate data, and associated tests of significance, are presented in Table 2.

**Table 2:** Comparison of demographics, deprivation and healthcare factors with OPAT

	Known to OPAT *(n = 729)* *n (%)*	*Not known to OPAT (n = 4215)* *n (%)*	Test of difference
Gender			
*Male*	457 (17%)	2185 (83%)	< 0.0001 ^a^
*Female*	272 (12%)	2030 (88%)	
Age Group			
*50 and under*	270 (17%)	1337 (83%)	< 0.0001 ^a^
*51 to 70*	321 (19%)	1327 (81%)	
*71 and over*	138 (8%)	1551 (92%)	
*Median age*	56	63	< 0.0001 ^b^
Deprivation			
*Most Deprived 1*	104 (12%)	741 (88%)	< 0.0001 ^a^
*2*	170 (13%)	1184 (87%)	
*3*	130 (15%)	756 (85%)	
*4*	104 (14%)	645 (86%)	
*Least deprived 5*	221 (20%)	889 (80%)	
Admissions (total over period)			
*Minimum*	1	1	
*Median*	1	1	
*Maximum*	12	9	
Co-morbidities (total over period)			
*Minimum*	0	0	0.0033 ^b^
*Median*^*3*^	2	3	
*Maximum*	63	72	
Bed days (total over period)			
*Minimum*	< 1	< 1	0.00035^b^
*Median total bed days*	3.3	4.0	
*Maximum*	504	1150	

^a^ Chi-Squared

^b^ Kruskal-Wallis

Although our aim was not to study the comparative effectiveness of OPAT treatment compared to inpatient care we did see evidence of a crude effect on length of inpatient stay over the period in those who were known to OPAT (median total bed days: 3.3 vs. 4.0, *p* = 0.00035).

**Fig. 2:**
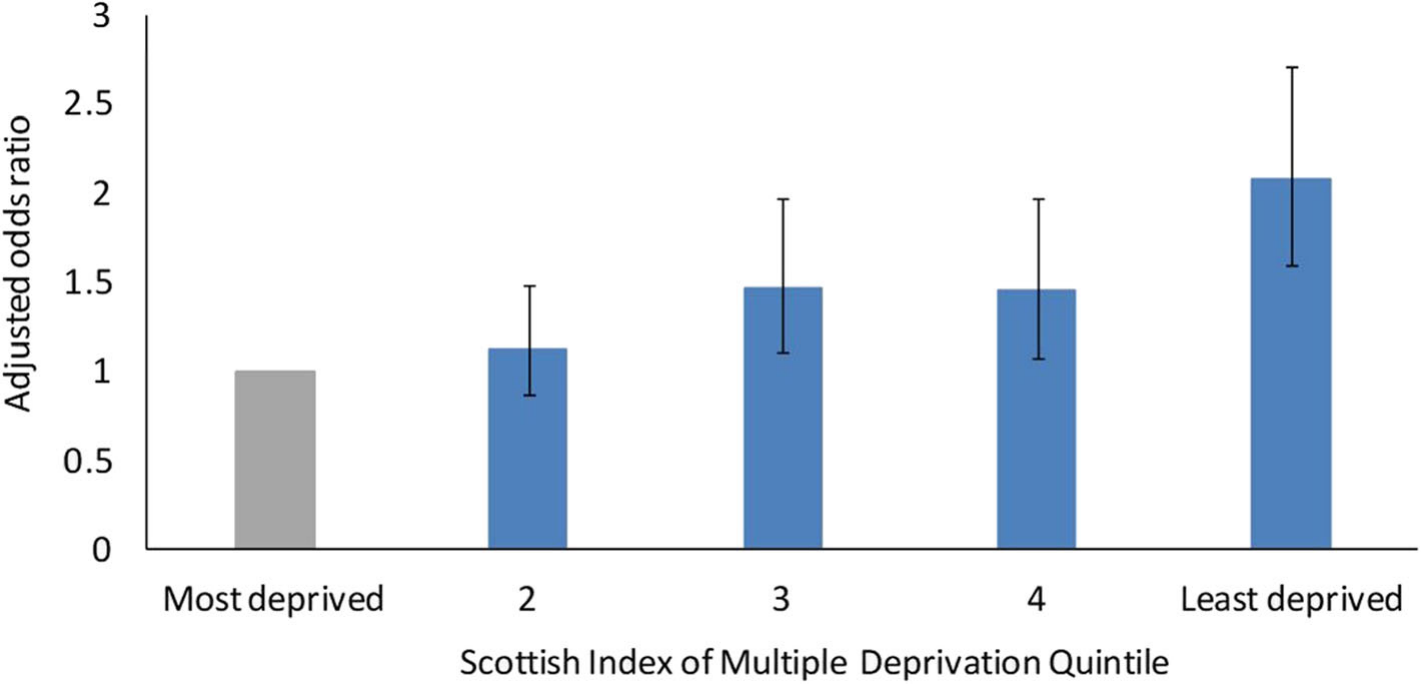
Adjusted^1^ odds ratio of OPAT referral stratified by SIMD quintile. ^1^ Adjusted for age, gender, time since first admission, distance to nearest OPAT clinic, total admissions, total co-morbidities and total length of stay over the period

An individual admitted due to cellulitis from the most deprived quintile was seven times more likely not to have been referred to OPAT than to have been referred. The adjusted odds ratio of being known to OPAT comparing the least to the most deprived quintile was 2.08 (95% CI: 1.60–2.71, *p* < 0.0001). This adjustment accounted for age, gender, distance to nearest OPAT clinic, time since first admission, total admissions, total co-morbidities and total length of stay over the period. These results are shown in Fig. 2, error bars show 95% confidence intervals.

A female patient admitted for cellulitis was 7.5 times more likely to not have been referred to OPAT than to have been referred. The adjusted odds ratio of being known to OPAT comparing female patients to male patients was 0.69 (0.58 to 0.82, *p* < 0.001), indicating men were 31% more likely to be known to the OPAT service. Again, this was adjusted for age, deprivation, distance to nearest OPAT clinic, total admissions, total co-morbidities and total length of stay over the period.

## Discussion

We have identified significant inequity in access to the OPAT service in a large Scottish health board, using hospitalisation due to cellulitis to probe this question. Although cellulitis admission is itself associated with deprivation we found that individuals admitted due to cellulitis from the most affluent quintile are more than twice as likely to have received an OPAT referral as those resident in the most deprived quintile. Men were also more likely to receive an OPAT referral than women. Both results were adjusted for: age; number co-morbidities; number of admissions and total length of stay; time since first admission; distance from OPAT clinic; and deprivation or gender as appropriate.

There are a number of limitations to this analysis. Cellulitis is not the only diagnosis for which OPAT is indicated however it represents the largest recorded reason for referral to OPAT in NHS Lothian constituting the majority (61%) of all referrals over the study period, and is reported to be the largest patient group for which OPAT is used in the UK [23]. By choosing to focus on this group we were able to focus the analysis and linkage, but lost the opportunity to study differences between admission reasons. It is possible that inequity is different in post-operative infections or other reasons for referral. Due to data limitations we cannot confirm that all included patients admitted for inpatient management of cellulitis received intravenous antimicrobials as an inpatient, though based on our clinical experience we contend that the majority would have. Specific reasons for non-referral to OPAT in this cohort are unknown, and some will relate to local exclusion criteria for the service, for example people who inject drugs and alcohol dependence, in addition to clinical instability making outpatient management inappropriate. An important goal of future work will be to disentangle appropriate reasons for non-referral from barriers that should be possible to overcome through adequate service provision, for example transportation.

Although data was linked individually, deprivation was assessed at a small geographic level using the SIMD. Whilst this is an imperfect measure of deprivation it was the only feasible metric to use, since additional data on individual situations (e.g. household income) were not available retrospectively.

There are several strengths to this analysis. The clinical data is matched at the individual level, we know whether these admitted individuals were individually known to the OPAT service and we have robust data on their distance from the OPAT clinic, SIMD score, gender, age and case complexity as indicated by their total number of admissions, lengths of stay and co-morbidities over a substantial time period. NHS admissions and referral data are high quality, collected by the health service for the purposes of performance management and national reporting, and quality assured by professionals.

Inequity in access may be driven by clinicians, some of whom may have a preference for inpatient management over community management of cellulitis. Clinicians may base their referrals on their own assessment of patient ability to utilise a service such as OPAT rather than clinical need. Other barriers may come from patients themselves. In previous studies patients have cited lack of access to transport and the cost of outpatient treatment as barriers to OPAT [24]. Ambulatory services require mobility, access to transport and time from work that those in deprived areas may be less able to afford. This may lead to either a tacit denial of offer by healthcare staff, or by a rejection of any proposed referral by the patient themselves.

The under-representation of women amongst OPAT referrals is unexplained. It is possible that women present more unwell with this condition rendering OPAT referral inappropriate, and certainly observational data has suggested that female gender is a risk factor for non-response to treatment at day three [25]. It has also been reported that female gender is associated with treatment failure amongst those that do get referred to OPAT [26]. However, the reasons behind these findings are unexplained and by not examining the intersection of gender, social health determinants and morbidity in the context of health service delivery we may be missing access inequity in under-represented groups [27]. More attention is needed in the design and implementation of health systems to ensure hidden inequities are exposed and reversed.

OPAT is described in the current UK guidelines as being a choice to be made by the patient, clinical situation allowing. Our evidence shows that without effort to ensure that OPAT is offered, and enablement to take advantage of it, this choice is far easier for some in our society. The guidelines make no mention of the measurement of equity of access as a quality indicator and based on our findings, this merits inclusion in further iterations [1].

Furthermore, best practice recommendations on reducing barriers to access are not available. We contend that further research on what works to improve equity is required with a particular focus on widening routes of referral, simplifying the referral process and ensuring patient transport to and from appointments is made as easy as possible. Previous evidence has shown that investment in these measures is likely to be cost-effective in the long-run but further evidence on this would also encourage implementation.

## Conclusions

Our analysis addresses an important and poorly researched issue: that the drive towards ambulatory in place of inpatient care may inadvertently widen healthcare inequity across social and gender divides. Our study was a quantitative exploration, further research is needed to untangle the reasons behind our findings, and what courses of action may remedy them.

As increasing pressure on healthcare services drives expansion of ambulatory care, it will be essential for service planners to be cognizant of the risk of inadvertently increasing inequity, and to ensure patients do not bear the brunt of cost savings achieved by secondary care.

This research would be easily replicable in other settings and services as the data is routinely available. It is unlikely OPAT is alone in suffering these inequities and robust assessment across outpatient services should be pursued across ambulatory care services. Equitable access to ambulatory services is essential to maximise their benefits to patients across social groups. As ambulatory care services such as OPAT expand, it is critical to identify and address the reasons underpinning inequality in access.

## Data Availability

The datasets generated and/or analysed during the current study are not publicly available as they comprise confidential identifiable patient level data. The data may be available from the corresponding author on reasonable request to persons suitably authorised to view the data.

## Abbreviations

OPAT: Outpatient Parenteral Antimicrobial Therapy
SIMD: Scottish Index of Multiple Deprivation
OR: Odds ratio
CI: Confidence Interval
NHS: National Health Service
UK: United Kingdom
ICD10: 10th revision of the International Statistical Classification of Diseases and Related Health Problems
CRN: Case Reference Number
EPR: Electronic patient record
